# An Observational Pilot Study using a Digital Phenotyping Approach in Patients with Major Depressive Disorder Treated with Trazodone

**DOI:** 10.3389/fpsyt.2023.1127511

**Published:** 2023-03-24

**Authors:** Jan Čermák, Slavomír Pietrucha, Alexander Nawka, Paola Lipone, Alessandro Ruggieri, Annalisa Bonelli, Alessandro Comandini, Agnese Cattaneo

**Affiliations:** ^1^Psychiatrie Říčany s.r.o., Říčany, Czechia; ^2^Centrum Duševního Zdraví—Psychiatrie s.r.o., Kutná Hora, Czechia; ^3^Institut Neuropsychiatrické Péče (INEP) (Psychiatric Outpatient Clinic), Praha, Czechia; ^4^Angelini Pharma S.p.A., Rome, Italy

**Keywords:** digital phenotyping, mental health, major depressive disorder, trazodone, active data collection, passive data collection

## Abstract

This 8-week study was designed to explore any correlation between a passive data collection approach using a wearable device (i.e., digital phenotyping), active data collection (patient’s questionnaires), and a traditional clinical evaluation [Montgomery-Åsberg Depression Rating Scale (MADRS)] in patients with major depressive disorder (MDD) treated with trazodone once a day (OAD). Overall, 11 out of 30 planned patients were enrolled. Passive parameters measured by the wearable device included number of steps, distance walked, calories burned, and sleep quality. A relationship between the sleep score (derived from passively measured data) and MADRS score was observed, as was a relationship between data collected actively (assessing depression, sleep, anxiety, and warning signs) and MADRS score. Despite the limited sample size, the efficacy and safety results were consistent with those previously reported for trazodone. The small population in this study limits the conclusions that can be drawn about the correlation between the digital phenotyping approach and traditional clinical evaluation; however, the positive trends observed suggest the need to increase synergies among clinicians, patients, and researchers to overcome the cultural barriers toward implementation of digital tools in the clinical setting. This study is a step toward the use of digital data in monitoring symptoms of depression, and the preliminary data obtained encourage further investigations of a larger population of patients monitored over a longer period of time.

## 1. Introduction

The complexity and heterogeneity of mental disorders has challenged psychiatry since the inception of the field (1). Commonly used diagnostic markers, such as blood tests, radiologic findings, and electrophysiological measurements can be inadequate to diagnose mental disorders accurately, and the severity of the symptoms (2). Advances in genetics and in neuroimaging offer new tools that psychiatry have embraced to advance understanding of the genetic and neural basis of psychiatric disorders (3). Even more recently, smartphones and wearable sensors/devices have been proposed as another set of tools for advancing understanding of physiological and behavioral perspectives of these disorders over time (4). Smartphone ownership is estimated to be 83.4% (5), and a willingness has been shown by patients to use a smartphone to monitor their mental health (6, 7). Growing literature in mental health points to the potential of smartphones and other personal digital devices to increase access to care (6), improve diagnosis, and enable remote monitoring (2, 8).

Termed “context sensing,” “personal sensing,” or “mobile sensing” by computer scientists, medicine has adopted the term “digital phenotyping” (9). Digital phenotyping, defined as the moment-by-moment quantification of the individual-level human phenotype *in situ* using data from smartphones and other personal digital devices, holds considerable potential for psychiatry, and the collection of phone-mediated social and behavioral markers may offer a new target for biological psychiatry (10). Digital phenotyping consists of the continuous acquisition of various metrics, including an individual’s physical activity and location, voice and speech patterns, and human-machine interactions, as sociability metrics (11, 12). By providing a highly granular view of these social and behavioral variables, mobile technology has the potential to transform current medical practice, reshape behavioral sciences, and improve the ability to deliver behavioral health treatments, thus creating a more holistic view of each patient’s trajectory in terms of mental and physical health (1, 9, 12–15).

Depression affects 322 million people worldwide and, in 2017, was considered the largest contributor to disability (16). According to the 5th edition of the Diagnostic and Statistical Manual of Mental Disorder (DSM-5) diagnostic criteria, major depressive disorder (MDD) is characterized by the presence of depressed mood or anhedonia for at least 2 consecutive weeks, during which abnormalities of neuro-vegetative function (appetite, weight loss, and sleep disturbance), psychomotor activity (e.g., loss of energy, agitation, or retardation), and cognition (feelings of worthlessness or inappropriate guilt), as well as anxiety and suicidal ideation, appear. Symptoms must be present most of the day and nearly every day (17). MDD is highly prevalent, with lifetime prevalence ranging from 2 to 21% (18), and considered one of the most disabling and burdensome mental illnesses (18, 19).

Depression is associated with several behavioral components (e.g., reduction in activity, psychomotor retardation, and changes in sleep), and motivational states (e.g., anhedonia), some of which may be detectable using a mobile phone or wearable device (20, 21). Recent technology has a significant potential for innovation to monitor behavioral and environmental risk indicators and to improve the long-term management and treatment of people suffering from depression (22).

Trazodone hydrochloride is a triazolopyridine derivative that was synthesized in Angelini Pharma laboratories in the 1960s. It is the first serotonin receptor antagonist and reuptake inhibitor developed for the treatment of depression (23). Trazodone is currently approved and marketed in several countries worldwide for the treatment of MDD, with or without anxiety, in adult patients. Relating to its pharmacological actions in humans, trazodone is defined as an up-to-date multimodal (24) and multifunctional drug with dose-dependent activity (25).

The trazodone once-a-day (OAD) formulation (available in 150 and 300 mg bisectable tablets) allows, by using a unique drug-delivery technology, release of the active ingredient over 24 h to improve treatment adherence and to provide an effective antidepressant dosing (300 mg/day) through a single administration. The pharmacokinetic profile of trazodone OAD is characterized by a slow increase of plasma level with a single low and delayed peak followed by a slow decline in plasma concentration, resulting in fewer associated adverse events (AEs) like sedation or hypotension (26).

Since its approval, the antidepressant efficacy and the favorable tolerability profile of trazodone have been confirmed in several pharmacological and clinical studies (27, 28). Results have demonstrated that trazodone was as effective as other antidepressant classes in the management of depressive disorders (26, 29–35). Contraindications for the use of trazodone OAD include known hypersensitivity to trazodone and any of the excipients, alcohol intoxication and intoxication with hypnotics, and acute myocardial infarction. The most common side effects include headache, dizziness, and sedation.

## 2. Methods

The aim of the study was to describe the clinical characterization of patients affected by MDD and treated with trazodone OAD monotherapy, applying the digital phenotyping approach. The study objectives and endpoints are presented in Table 1.

**Table 1:** Study objectives and endpoints.

Primary objective	Primary endpoint
To describe the clinical characterization of patients through passive data collected during the 8-week observation period	The trend of passive parameters over time. The following variables were considered passive data: distance traveled (in meters), step count, calories burned (in kilocalories), duration of deep sleep period (in hours), duration of light sleep period (in hours), duration of sleep (in hours), duration of awake sleep period (in hours), and wake-up count.
Secondary objectives	Secondary endpoints
To describe the clinical characterization of patients through passive data collected during the 8-week observation period^a^	Trend of sleep score over time
To describe the clinical characterization of patients by active data collected during the 8-week observation period	Trend of active parameters over time. The active parameters were the following domains of the patient rating scale through the web-based platform: depression, sleep, warning signs, anxiety, and medication intake.
To explore the relationship between passive data and traditional metrics collected in-clinic (MADRS) over the 8-week observation period	Relationship between MADRS score and passive data measurements
To explore the relationship between active data and traditional metrics collected in-clinic (MADRS) over the 8-week observation period	Relationship between MADRS score and active data measurements
To explore the relationship between passive and active data over the 8-week observation period	Relationship between passive and active measurements

MADRS, Montgomery-Åsberg Depression Rating Scale.

^a^The trend of sleep score over time was a secondary endpoint relating to the primary objective.

### 2.1. Study design

This was an observational, prospective, single group, multicenter, pilot study in patients affected by MDD and treated with trazodone OAD monotherapy, which applied the digital phenotyping approach to clinical characterization. The study was conducted at six private psychiatric practices in the Czech Republic, three of which enrolled patients in the study.

#### 2.1.1. Inclusion and exclusion criteria

Only outpatients with a diagnosis of MDD (according to the DSM-5 or International Classification of Diseases) and judged by a physician as eligible to start a pharmacological treatment with trazodone OAD monotherapy were included in the study. The decision to start trazodone OAD monotherapy must have been made prior to the patient’s enrollment in the study and was made independently from the physician’s decision to include the patient in the study.

Patients who met any of the contraindications to the administration of trazodone OAD monotherapy according to the approved summary of product characteristics were excluded from the study, and trazodone OAD was the only antidepressant permitted for treatment of MDD during the study observation period. Due to the real-world conditions of the study, dosing and treatment duration of trazodone OAD monotherapy were at the discretion of the physician, in accordance with local clinical practice, local labeling, and the patient’s medical needs.

#### 2.1.2. Data collection

Study assessments and data collection occurred during routine visits within the normal course of care at Baseline (Day 0) and after approximately 8 weeks at the routine Follow-up visit. At Baseline, after a patient’s enrollment, historical data were collected from their medical chart or, if this was not available, directly from the patient during the visit. Data collected from each patient included:

- Demographic data including marital status, occupational status, and educational level.
- Medical history, including the date of MDD diagnosis.
- Physical examination focusing on existing signs and symptoms.
- Current episode of MDD (i.e., start date and recurrence).
- Current antidepressant therapy (i.e., start date of trazodone OAD monotherapy and current dose).
- Montgomery-Åsberg Depression Rating Scale (MADRS).
- Previous (only drugs used for treatment of depression) and concomitant treatments (pharmacological and non-pharmacological treatments, including psychotherapy and device).

In addition, patients were provided with a digital wearable device which, together with the associated web-based platform (see Section 2.1.2.1), was activated at Baseline.

At the routine Follow-up visit 8 weeks after Baseline, MADRS was completed, and patients were asked about any change in antidepressant therapy and concomitant treatments, and the occurrence of any AEs since their last visit. The web platform and wearable device were deactivated, and the wearable device was returned to the physician at this visit.

If any unscheduled visits occurred during the 8-week observation period, any data regarding the patient’s psychiatric status, any changes in antidepressant therapy (newly assigned antidepressant drug, dose, and reason for discontinuation/switch/augmentation), changes in concomitant treatments and AEs, and any other information deemed relevant by the physician for the purpose of the study were collected in the patient’s medical chart during the visit.

The protocol, the patient information sheet/informed consent form, the personal data processing notice (PDPN), and any information provided to patients were approved by an independent ethics committee prior to each study center’s initiation. The study was conducted in accordance with the Declaration of Helsinki, Ethical Principles for Medical Research Involving Human Patients, applicable Good Clinical Practice, and Good Pharmacoepidemiology Practice principles. Written informed consent was obtained from all patients prior to enrollment into the study, as dictated by the Declaration of Helsinki. A copy of the signed consent, including patient’s information and a copy of the PDPN, was given to the patient, together with any needed clarification. Sufficient time was given to enable the patient to take a decision whether to participate in the study.

##### 2.1.2.1. Passive data collection

Once eligibility to take part in the study was confirmed, the patient was provided with a digital wearable device, i.e., a smartwatch (Withings Move ECG) which was to be worn all day, including at night, for the entire observation period. The smartwatch was activated by the physician at Baseline (Day 0), and the following passive data were automatically recorded by the smartwatch: distance traveled, steps, calories, sleep duration, sleep phases, sleep regularity, sleep interruptions, and sleep quality. Additionally, a “sleep score” was derived directly in the tracking device from the passively measured sleep data. The sleep score measured every night’s sleep and provided a score out of 100 points based on four key inputs: duration (total time spent sleeping); depth (part of night spent in restorative phases and deep sleep); regularity (consistency between your bed- and rise-times); and interruptions (time spent awake). Higher scores indicated a better sleep quality ranging from 1 (worst sleep quality) to 100 (best sleep quality).

Patients installed the “Health Mate” app on their smartphone to collect the data recorded by the smartwatch. A web-based platform, “MyHealth,” was provided by DataRiver and activated by the physician at Baseline (Day 0). Patients were asked to save the web platform page as if it were an application (app) on their smartphone at Baseline (Day 0). Data collected by Health Mate synchronized with the “MyHealth” portal provided by DataRiver. Access to MyHealth was granted only to users activated and authenticated on the web application by site staff, in the role of the “coach.” Site staff could also assign questionnaires to patients using the “coach” role. Patients were set up with the role of “subject” and could log in to MyHealth to complete assigned questionnaires. After performing user acceptance testing, the system was validated by DataRiver prior to the system going live.

##### 2.1.2.2. Active data collection

MyHealth, on the study patients’ smartphone, was used by study patients to complete a weekly patient questionnaire. Patients received a text alert when a new questionnaire was due, which was to be completed within 7 days of receiving the text alert.

The collection of active data was through the completion of the online questionnaire directly by the patient every week during the 8-week observation period. This questionnaire asked, “*In the past week, have you been bothered by the following problems?*,” and collected the patient’s feedback on items relating to the following domains:

**Table:** 

Depression: Little interest or pleasure in doing things Feeling down, depressed, or hopeless Withdrawing from social interaction Poor appetite or overeating	Sleep: Difficulty staying asleep Difficulty falling asleep Waking up too early Do not feel rested after waking up
Warning signs Feeling tired Trouble concentrating Feeling confused or puzzled	Anxiety Feeling nervous, scared, or anxious Trouble relaxing Unable to cope with stress Worrying too much
Medication intake Missing doses of medication	Easily annoyed or irritated

Patients rated each item from 0 (not at all) to 3 (frequently) depending on how they felt during the previous week. Scores were summed for each domain, with higher values indicating a worse outcome.

##### 2.1.2.3. In-clinic data collection: Montgomery-Åsberg depression rating scale

The Montgomery-Åsberg Depression Rating Scale (MADRS) was developed in 1979, and is a widely used clinician-rated measure of depressive severity (36, 37). MADRS was completed on a specific paper form by the physician at Baseline (Day 0) and at the routine Follow-up visit (approximately 8 weeks from Baseline), or at an unscheduled visit in case of premature withdrawal from the study. The physician followed a structured interview relating to the patient’s depression over the previous week. Ten items (reported sadness, apparent sadness, inner tension, reduced sleep, reduced appetite, concentration difficulties, lassitude, inability to feel, pessimistic thoughts, and suicidal thoughts) were scored individually from 0 to 6 and summed to give the total MADRS score. MADRS scores ranged between 0 and 60, with higher scores indicating a worse depression.

#### 2.1.3. Data analysis

##### 2.1.3.1. Analysis sets

The following analysis sets were defined:

- Safety population: all patients with informed consent signed who received any amount of trazodone OAD.
- Modified intent-to-treat population (mITT): all patients from the Safety population who additionally had at least 1 day with tracked passive data per week during at least four study weeks.
- Per protocol (PP): all patients from the Safety population with tracked passive data during all days of the study period (from Baseline visit to Follow-up visit) and who met all eligibility criteria.

##### 2.1.3.2. Primary endpoint methodology

In order to analyze the primary endpoint, the trend of passive parameters, a graphical representation of each parameter value per patient and study day was presented through scatter plots. In these plots, the mean value of all patients at each study day and the loess (locally weighted scatter plot smoothing) regression line were included. Additionally, summary statistics, including the 95% CI of the mean of each parameter per study day, were tabulated. All analyses were performed for the mITT population.

The first sensitivity analysis was a repeat of the previous analysis (both table and plot) for the PP population. As a second sensitivity analysis, all passive parameters were summarized by the average of study week, including change from Week 1 and a similar graphic for the primary endpoint was used representing the study week values. This analysis is presented for the mITT and PP populations.

##### 2.1.3.3. Secondary endpoint methodology

###### 2.1.3.3.1. Trend of sleep score over time

In order to analyze these data, a graphical representation of the sleep score per patient and study day was presented through scatter plots, similarly to the primary endpoint analysis. Additionally, summary statistics per study day were tabulated. The same analysis (scatter plot and tabulation) was done by the average of study week. All these analyses were performed in the mITT population.

The sleep score measured every night’s sleep and provided a score out of 100 points based on four key inputs: duration (total time spent sleeping); depth (part of night spent in restorative phases and deep sleep); regularity (consistency between your bed- and rise-times); and interruptions (time spent awake). Higher scores indicated a better sleep quality ranging from 1 (worst sleep quality) to 100 (best sleep quality). Sleep score was derived directly in the tracking device.

###### 2.1.3.3.2. Trend of active parameters over time

A graphical representation of each parameter per patient and study week was presented through scatter plots, including all patient means and a regression line. Summary statistics of each parameter per study week were tabulated and each individual item was also categorically described. A decrease in score for each of the active parameters corresponded to an improvement in clinical outcome. All these analyses were performed in the mITT population.

###### 2.1.3.3.3. Relationship between MADRS score and passive data measurements

Montgomery-Åsberg Depression Rating Scale score is a traditional metric, with scores ranging from 0 to 60 (higher scores indicate worse depression), which was collected in clinic at Baseline and after 8 weeks. A descriptive summary of MADRS score per visit and change from Baseline (CFB) was tabulated. Spearman’s correlation between MADRS score at Baseline and each passive parameter at Week 1 were summarized. The same analysis was repeated between MADRS at the Follow-up visit with each passive parameter at Week 8, and between CFB MADRS at Follow-up with change from Week 1 at Week 8 with each passive parameter. A scatter plot for each passive parameter displaying the values of passive data and MADRS score per analyzed time point is presented. These plots included the corresponding correlation value. Finally, a panel representing the level of correlation using colors by time point and parameter was produced. All analyses were performed in the mITT population.

###### 2.1.3.3.4. Relationship between MADRS score and active data measurements

A similar analysis to that for the relationship between MADRS score and passive data measurements was performed, including Spearman’s correlations and scatter plots and color panel between MADRS score and each active parameter (scale dimension scores) per analyzed time point (Week 1/Baseline and Week 8/Follow-up).

###### 2.1.3.3.5. Relationship between passive and active measurements

Spearman’s correlations between each active and passive parameter per study week were provided. Additionally, per each combination of active and passive parameter, a panel of scatter plots for each study week was provided. All analyses were done in the mITT population.

## 3. Results

### 3.1. Study patients

Eleven patients were enrolled in and completed the study. Ten (90.9%) patients who signed the informed consent form and received any amount of trazodone OAD had at least 1 day with tracked passive data per week during at least 4 study weeks, and so were included in the mITT population. One patient did not complete the registration for passive data collection on the wearable device, and so was not included in the mITT population. Demographic and baseline characteristics of the mITT population are provided in Table 2.

**Table 2:** Demographic and Baseline Characteristics (mITT Population).

Variable/Category	Overall (*N* = 10)
Age (years)	
*N*	10
Mean (standard deviation)	36.9 (14.58)
Median	35.0
Quartile 1, quartile 3	23.0, 45.0
Minimum, maximum	22, 65
Sex	
Male	3 (30.0%)
Female	7 (70.0%)
Race	
White	10 (100%)
Education detail	
Primary education	3 (30.0%)
Secondary education	5 (50.0%)
University or higher	2 (20.0%)
Occupational status	
Employed full-time	8 (80.0%)
Employed part-time	1 (10.0%)
Retiree	1 (10.0%)
Marital status	
Single	5 (50.0%)
Married	2 (20.0%)
Divorced	1 (10.0%)
Separated	1 (10.0%)
Engaged	1 (10.0%)
Time since MDD diagnosis (years)	
*n*	10
Mean (standard deviation)	4.551 (5.9095)
Median	1.936
Quartile 1, quartile 3	0.019, 10.004
Minimum, maximum	0.00, 15.17
Time since onset of current MDE (years)	
*N*	10
Mean (standard deviation)	1.824 (2.0268)
Median	1.392
Quartile 1, quartile 3	1.02, 1.704
Minimum, maximum	0.24, 7.44
MDE recurrence	
Single	4 (40.0%)
Recurrent	6 (60.0%)
Medical and psychiatric history	
Patients reporting at least 1 medical/psychiatric history	3 (30.0%)^a^
Prior and concomitant treatments	
Patients reporting taking at least 1 prior medication	9 (81.8%)^b^
Patients reporting taking at least 1 concomitant medication	4 (36.4%)^c^

MDD, major depressive disorder; MDE, major depressive episode; Q, quartile; SD, standard deviation; and SSRIs, selective serotonin reuptake inhibitors.

^a^Type 2 diabetes mellitus, anxiety, social anxiety disorder, asthma, and hypertension were each reported by one patient.

^b^SSRIs (taken by five patients; 45.5%) and other antidepressants (taken by four patients; 36.4%) were the most common prior medications reported. Antiandrogens and estrogens; benzodiazepine derivatives; beta blocking agents, selective; progestogens and estrogens, fixed combinations; and selective beta-2-adrenoreceptor agonists were each taken by one patient.

^c^Antiandrogens and estrogens; beta blocking agents, selective; progestogens and estrogens, fixed combinations; selective beta-2-adrenoreceptor agonists; and SSRIs were each taken by one patient.

Device activation and inactivation was carried out, as scheduled, at Baseline and the Follow-up visits, respectively, for all 11 patients (100.0%).

The majority of dose adjustments, recorded in eight patients (72.7%), were standard titrations where the dose of trazodone OAD was increased in a manner consistent with that specified in the study protocol. No dose adjustment was made during the study for two patients, who remained on a dose of 75.0 mg/day, which was below the therapeutic dose level.

The median dosage of trazodone OAD monotherapy at Day 0 was 75.0 (range: 75.0–150.0) mg/day; 75.0 mg/day was the starting dose for nine of the 11 patients (81.8%). The starting dose of trazodone OAD for the remaining two patients (18.2%) was 150 mg/day. The majority of patients (eight patients, 80.0%) had changes to their treatment during the study, all of which were at scheduled visits. The maximum dose reached was 300.0 mg/day in two patients; 225.0 mg/day in one patient; 150.0 mg/day in six patients; and two patients remained on a 75.0 mg/day dose for the duration of the study.

### 3.2. Primary endpoint

**Figure 1:**
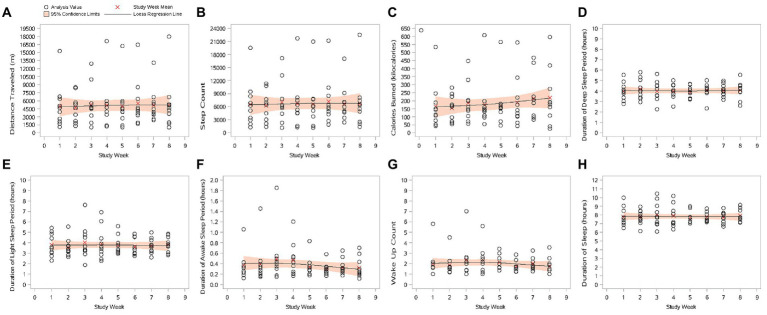
Scatter plots of passive data by study week (mITT Population). **(A)** Distance traveled (m); **(B)** step count; **(C)** calories burned (kilocalories); **(D)** duration of deep sleep period (hours); **(E)** duration of light sleep period (hours); **(F)** duration of awake sleep period (hours); **(G)** wake-up count; and **(H)** duration of sleep. Study Week 1 lasted from Study Day 1 to Study Day 7. The following weeks are multiples of 7 study days. The plotted value is the average of each study week. mITT Population = all patients from the Safety population who additionally have at least 1 day with tracked passive data per week during at least 4 study weeks.

Scatter plots showing the trend of passive data by study week are shown in Figure 1.

#### 3.2.1. Distance traveled

Mean (SD) daily distance traveled per day during Week 1 was 4949.6 (4098.86) m. Except for a reduction in mean (SD) daily distance traveled from Week 1 during Week 2 (−257.8 [2565.42] m), daily distance traveled during all other weeks was greater than that during Week 1. A peak in mean (SD) distance traveled per day was recorded at Week 6 (5495.5 [4348.06] m); a mean (SD) increase from Week 1 of 545.8 (3261.56) m.

#### 3.2.2. Step count

Mean step count per day during Week 1 was 6526.1 (5312.00) steps. As with distance traveled, there was a reduction in the mean (SD) daily step count from Week 1 during Week 2 (−315.0 [3307.55] steps); daily step count during all other weeks was greater than that during Week 1. A peak in daily step count was recorded at Week 6 (7165.2 [5526.07] steps); a mean (SD) increase from Week 1 of 639.0 (4185.37) steps per day.

#### 3.2.3. Calories burned (kilocalories)

Mean (SD) daily calories burned during Week 1 was 165.0 (144.15) kcal. The mean (SD) daily number of calories burned during Week 2 was less than that during Week 1 (a reduction of −11.0 [100.86] kcal). An increase from daily calories burned during Week 1 was seen during all other weeks, with a peak at Week 8 of 218.3 (174.77) kilocalories burned per day; an increase of 53.3 (144.79) kcal from Week 1.

#### 3.2.4. Sleep parameters (deep sleep, light sleep, time awake, wake-up count, and duration of sleep)

None of the five passively collected sleep parameters showed any clear trends after starting treatment with trazodone OAD monotherapy. Changes from Week 1 in mean (SD) sleep durations were small; during the 8 weeks of the study, the biggest decrease (at Week 2, in duration of light sleep) was 0.2 (1.00) h, and the biggest increase (at Week 3, in duration of sleep) was 0.3 (1.43) h, both of which equate to around 15 min. Similarly, changes from Week 1 in the mean (SD) wake-up count were small; the biggest mean (SD) decrease from Week 1 was −0.3 (1.03) times at Week 6; the biggest mean (SD) increase from Week 1 was 0.4 (0.40) times at Week 4. Such quantitively small and inconsistent changes would not be considered clinically meaningful.

### 3.3. Secondary endpoints

#### 3.3.1. Trend of sleep score over time

**Figure 2:**
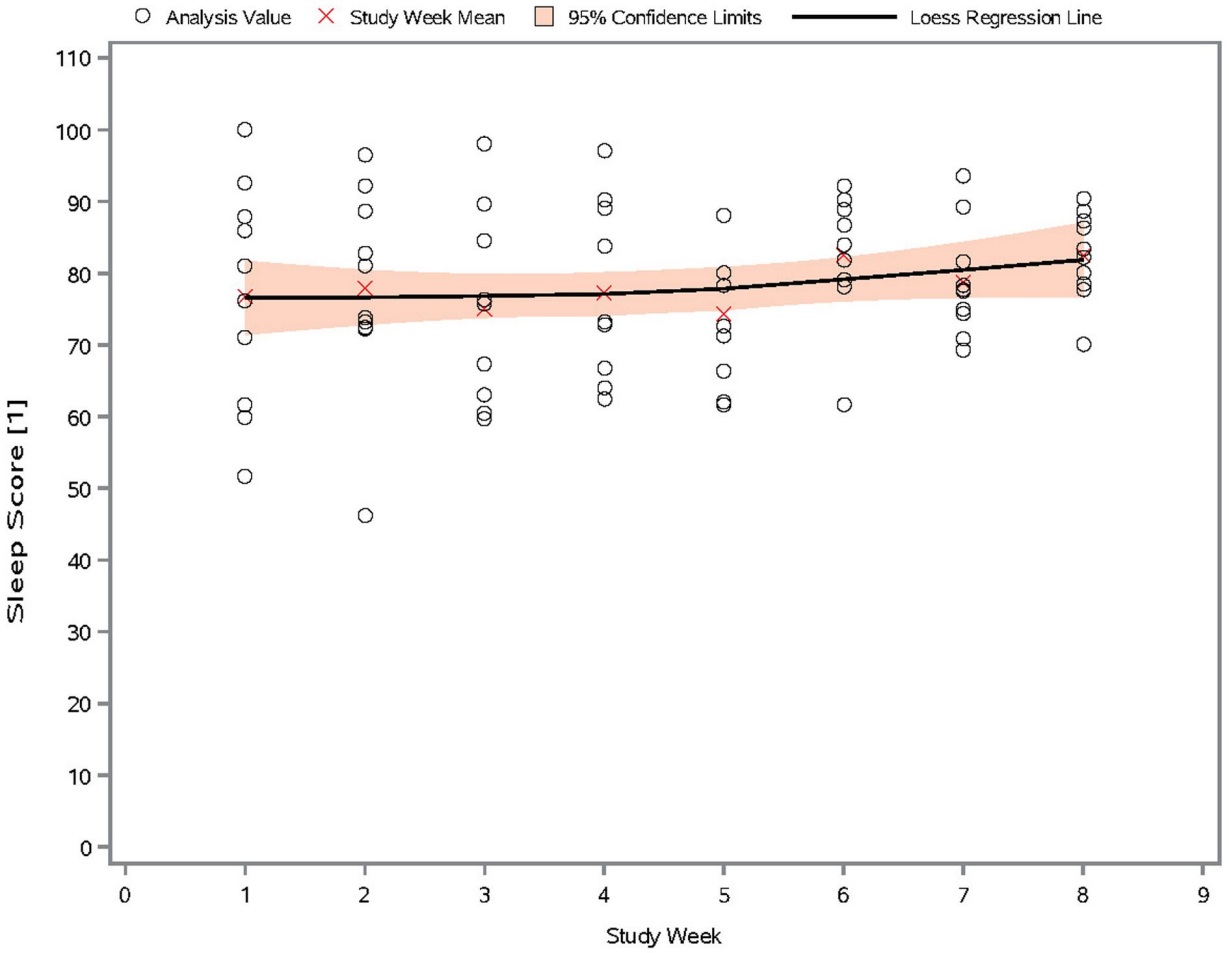
Scatter plot of sleep score by study week (mITT Population). The sleep score measures sleep quality, and ranges from 0 to 100. Higher scores indicate better sleep quality.

A scatter plot of sleep score data, measured passively and derived directly in the tracking device, in the mITT population is provided in Figure 2. During the 8 weeks of the study, there was an overall increase in mean (SD) sleep score, from 76.8 (15.60) during Week 1 to 82.4 (6.14) during Week 8, indicating a gradual improvement in sleep quality.

#### 3.3.2. Trend of active parameters over time

**Figure 3:**
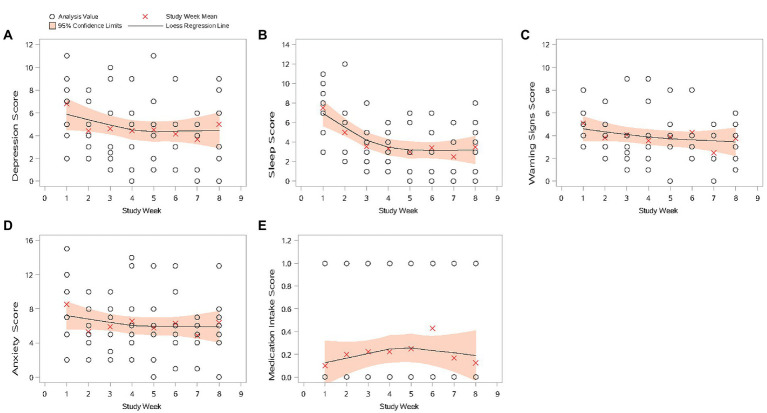
Scatter plots of active data by study week (mITT Population). **(A)** Depression score; **(B)** sleep score; **(C)** warning signs score; **(D)** anxiety score; and **(E)** medication intake score. If there were no missing active data, the surveys were assigned to study weeks chronologically. Otherwise, Study Week 1 was assigned for the assessment collected at target date Study Day 1 ±3 days. The following study weeks are assigned in multiples of 7 days (with ±3 days at end date as window) from Study Week 1. All scale dimensions scores are the sum of the corresponding items with higher values indicating a worse outcome. mITT Population = all patients from the Safety population who additionally have at least 1 day with tracked passive data per week during at least four study weeks.

An improvement in clinical outcome (corresponding to a decrease in score) was recorded for all active data parameters, except for medication intake score, which showed an overall increase in the number of times a dose of medication had been missed during the study (Figure 3). However, this equated to 10.0% of patients indicating they “sometimes” missed a dose of medication at Weeks 1, 7, and 8; 20.0% of patients at Weeks 2 through 5; and 30.0% of patients at Week 6; all other patients at all weeks indicated doses of medication were missed “not at all.”

For mean depression, sleep scores, warning signs, and anxiety scores, a decrease from Week 1 was recorded at Week 2, and scores remained generally similar through Week 8. Week 7 was the exception, when the lowest mean scores during the 8 weeks of the study were recorded for these parameters.

#### 3.3.3. Relationship between MADRS score and passive data measurements

In the mITT population, mean (SD) MADRS score at Baseline was 27.7 (5.72). After the 8 weeks of the study, mean (SD) MADRS score was 12.5 (9.16), a mean (SD) reduction of 15.2 (7.80), indicating a decrease in the extent of the symptoms of depression during the study.

**Figure 4:**
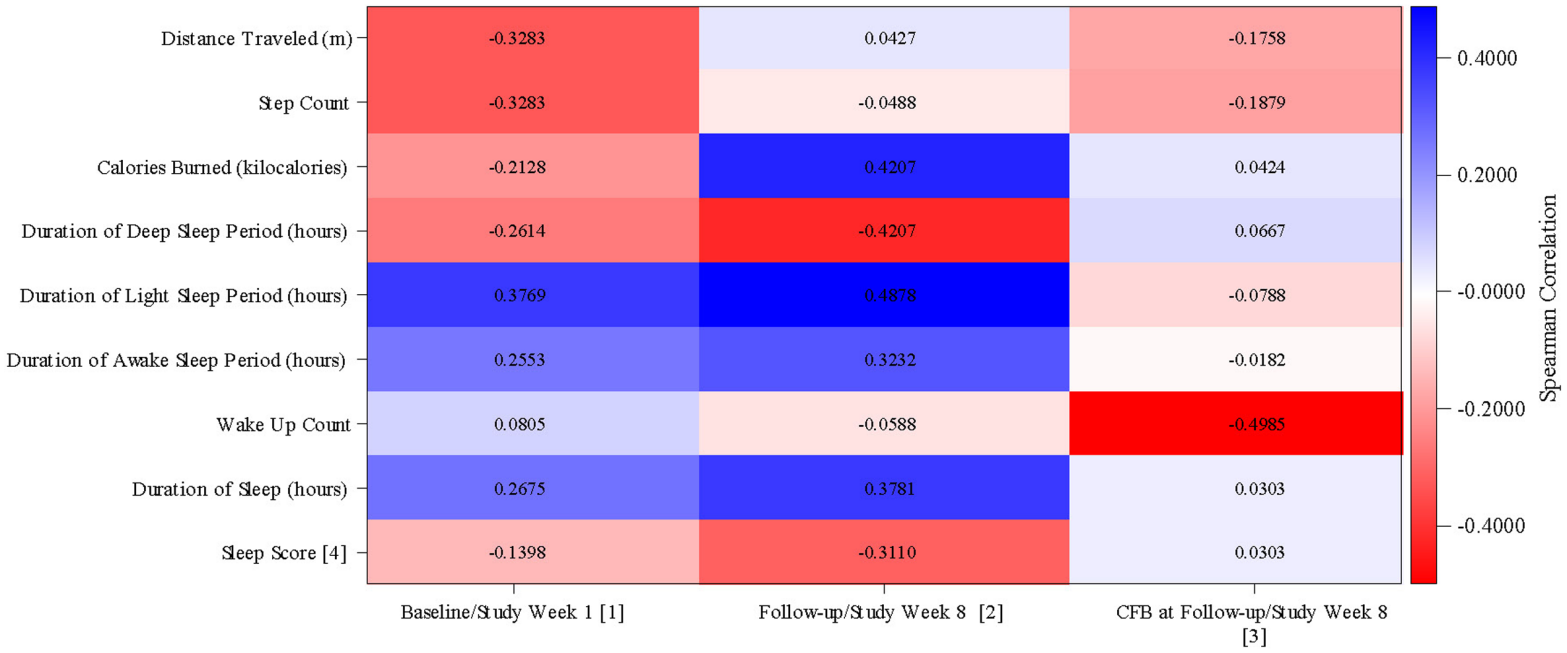
Correlation between passive data and MADRS score (mITT Population). MADRS, Montgomery Äsberg Depression Rating Scale; CFB, Change from Baseline. (1) Correlation between MADRS score at Baseline (defined as the value obtained in the Day 0 visit) and averaged passive data from Study Week 1. (2) Correlation between MADRS score at Follow-up and averaged passive data from Study Week 8. (3) Correlation between MADRS score change from baseline at Follow-up and averaged passive data change from Study Week 1 from Study Week 8. (4) The sleep score which measures sleep quality, and ranges from 0 to 100. Higher scores indicate better sleep quality. mITT Population = all patients from the Safety population who additionally have at least 1 day with tracked passive data per week during at least 4 study weeks.

Figure 4 shows the correlations between the passive data measurements and MADRS score. Data used are the average passive data from Week 1 and Week 8 and MADRS scores obtained at Baseline and the Follow-up visit. Higher MADRS scores indicate worse depression. A Spearman’s correlation ≥0.7 was considered a good correlation.

There were negative correlations between the duration of the deep sleep period (Spearman’s correlation = −0.26) and the sleep score (Spearman’s correlation = −0.14) and MADRS score at Baseline. Both correlations were stronger at Week 8 (deep sleep period: Spearman’s correlation = −0.42; sleep score: Spearman’s correlation = −0.31); better sleep quality, as indicated by a longer duration of deep sleep and a higher sleep score, at Week 8 correlated with a lower MADRS score.

There were positive correlations between the durations of the light sleep period (Spearman’s correlation = 0.38), the awake sleep period (Spearman’s correlation = 0.26), and the duration of sleep (Spearman’s correlation = 0.27) and MADRS score at Baseline. All correlations were stronger at Week 8 (light sleep period: Spearman’s correlation = 0.49; awake sleep period: Spearman’s correlation = 0.32; sleep duration: Spearman’s correlation = 0.38). A shorter duration of light sleep, time spent awake, and sleep overall at Week 8 was correlated with a lower MADRS score.

At Baseline, the correlation between calories burned and MADRS was weakly negative (Spearman’s correlation = −0.21), with a higher number of calories burned correlated with a lower MADRS score. At Week 8, this correlation was reversed (Spearman’s correlation = 0.42), with a lower number of calories burned correlated with a lower MADRS score.

Distance traveled and step count (for both, Spearman’s correlation = −0.33) were negatively correlated with MADRS score at Baseline. At Week 8, distance traveled showed a marginally positive correlation with MADRS score (Spearman’s correlation = 0.04). The correlation between step count and MADRS score at Week 8 remained negative, but marginally so (Spearman’s correlation = −0.05).

The correlation between wake-up count and MADRS score at Baseline was marginally positive (Spearman’s correlation = 0.08); at Week 8, the correlation was marginally negative (Spearman’s correlation = −0.06); little change was seen after 8 weeks of treatment.

#### 3.3.4. Relationship between MADRS score and active data measurements

**Figure 5:**
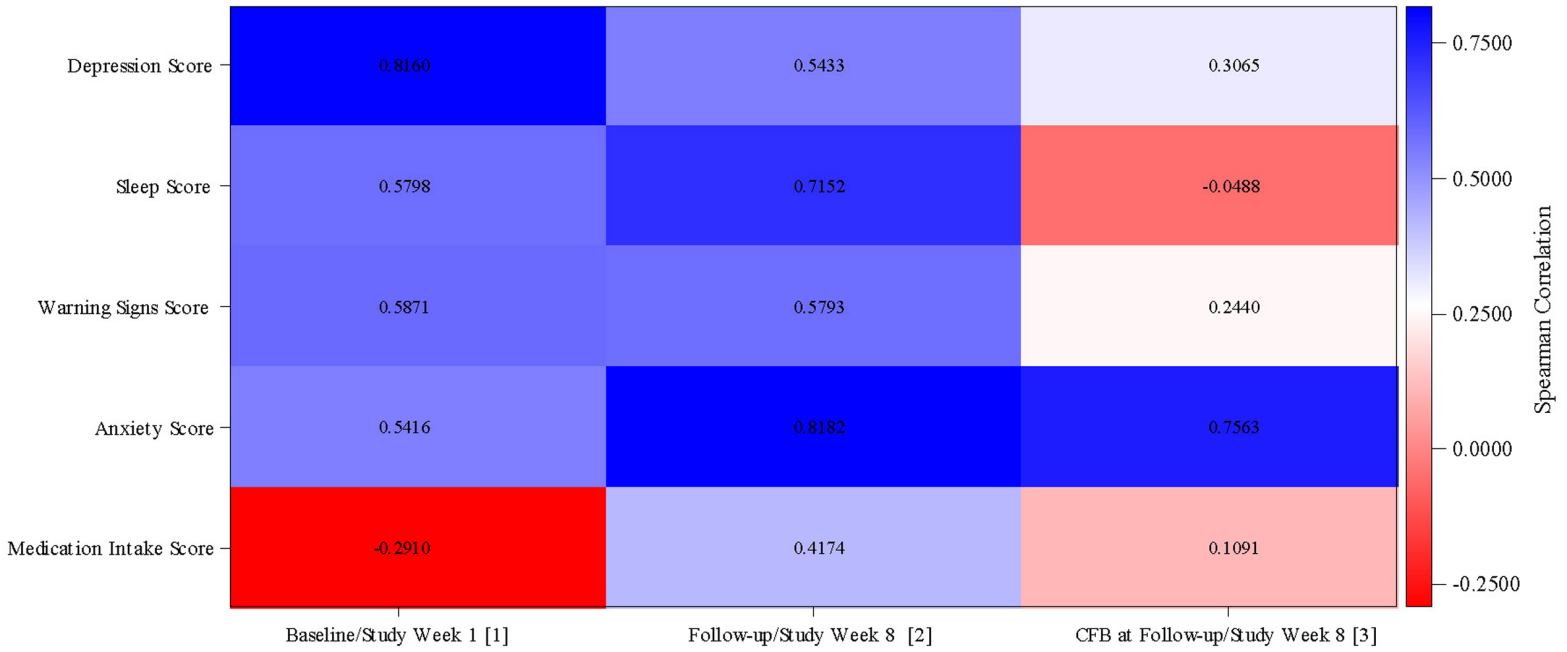
Correlation between active data and MADRS score (mITT Population). MADRS, Montgomery Äsberg Depression Rating Scale; CFB, Change from Baseline. (1) Correlation between MADRS score at Baseline (defined as the value obtained in the Day 0 visit) and active data from Study Week 1. (2) Correlation between MADRS score at Follow-up and active data from Study Week 8. (3) Correlation between MADRS score change from baseline at Follow-up and active data change from Study Week 1 from Study Week 8. mITT Population = all patients from the Safety population who additionally have at least 1 day with tracked passive data per week during at least 4 study weeks.

Figure 5 shows the correlations between the active data measurements and MADRS Score. Data used are the average active data from Week 1 and Week 8 and MADRS scores obtained at Baseline and the Follow-up visit. Higher MADRS scores indicate worse depression; for active data, higher scores indicate a worse outcome. Again, a Spearman’s correlation ≥0.7 was considered a good correlation.

Sleep score (Spearman’s correlation = 0.58) and anxiety score (Spearman’s correlation = 0.54) were both positively correlated with MADRS score at Baseline; at Week 8, both showed a stronger positive correlation with MADRS score (sleep score: Spearman’s correlation = 0.72; anxiety score: Spearman’s correlation = 0.82). After 8 weeks of treatment, there was a stronger correlation, with high sleep scores and high anxiety scores corresponding to worse depression.

The strength of the positive correlation between the depression score and MADRS score decreased from Baseline (Spearman’s correlation = 0.82) to Week 8 (Spearman’s correlation = 0.54); a high depression score still correlated with a high MADRS score after 8 weeks of treatment, but not as strongly. There was little change in the correlation between the warning signs score and MADRS score between Baseline (Spearman’s correlation = 0.59) and Week 8 (Spearman’s correlation = 0.58).

At Baseline, there was a negative correlation between the medication intake score and MADRS score (Spearman’s correlation = −0.29). Following 8 weeks of treatment, this correlation was positive (Spearman’s correlation = 0.42), with a higher medication intake score (i.e., lower compliance) correlated with a higher MADRS score.

#### 3.3.5. Relationship between passive and active measurements

It was difficult to determine any clear relationships between passive and active measurements (Supplementary Table 1). For each relationship, the direction of the correlations (the majority of which were weak) was not constant during the 8 weeks of the study.

The most consistent (relating to direction only; the strength of the correlations was variable) correlations were between:

- The duration of the deep sleep period (passive) and sleep score (active), where a negative Spearman’s correlation was recorded at each time point; a longer duration of deep sleep correlated with a lower (better outcome) sleep score
- The duration of the deep sleep period (passive) and warning signs score (active), where a negative Spearman’s correlation was recorded at each time point except for Week 5; a longer duration of deep sleep correlated with a lower (better outcome) warning signs score
- The duration of the light sleep period (passive) and depression score (active), where a positive Spearman’s correlation was recorded at each time point; a longer duration of light sleep correlated with a higher (worse outcome) depression score
- The duration of the light sleep period (passive) and sleep score (active), where a positive Spearman’s correlation was recorded at each time point except for Week 4; a longer duration of light sleep correlated with a higher (worse outcome) sleep score
- The duration of the light sleep period (passive) and anxiety score (active), where a positive Spearman’s correlation was recorded at each time point except for Week 4; a longer duration of light sleep correlated with a higher (worse outcome) anxiety score
- The duration of sleep (passive) and depression score (active), where a positive Spearman’s correlation was recorded at each time point except for Week 8; a longer duration of sleep correlated with a higher (worse outcome) depression score

## 4. Discussion

This observational study aimed to explore the potential of the digital phenotyping approach in describing the clinical course and outcome in patients with MDD treated with trazodone OAD monotherapy. During this 8-week study, the clinical characterization of patients affected by MDD was described using three methods: digital phenotyping (passive data collection by smartwatch), active data collection (completion of an online survey by the patient), and traditional in-clinic data collection (completion of the MADRS by the investigator). The description of functional parameters, collected passively, and the relationship between these data with data collected actively (ePRO) and the clinical evaluation was also explored. The implementation of the digital phenotyping method, together with traditional in-clinic data collection, aimed to engage patients’ active participation to the study (ePRO), to minimize the burden of in-clinic visits and to explore the potential of this hybrid approach to better monitor the clinical evaluation of the disease, and treatment effectiveness.

Due to a low rate of enrollment, 11 out of 30 planned patients were included in this study, with 10 patients contributing to passive data collection and thus being included in the analysis. The small size of the population hinders the conclusions that can be drawn from this study, particularly concerning passive data, which are characterized by the high variance in the measured variables. The use of the hybrid (digital and clinical) approach in this study was expected to facilitate patient participation and engagement; however, many patients were unexpectedly skeptical about the use of the wearable device and did not feel confident with the digital technology, leading to early termination of enrollment.

Previous studies have clearly established the efficacy of trazodone OAD in patients with MDD, with significant reduction of MADRS scores and improvements in the quality of sleep among the benefits of treatment (26–28, 35). Despite the low number of patients, data obtained *via* the three methods employed in this study, i.e., passive data collection, active data collection, and traditional in-clinic data collection, allowed a preliminary digital and clinical phenotyping of patients treated with trazodone OAD monotherapy.

Traditional in-clinic efficacy evaluation (MADRS score), data collected actively (with the exception of the medication intake score), and the sleep score (derived from passively collected data) were coherent and indicated a decrease in the extent of the symptoms of depression following the start of monotherapy with trazodone OAD, confirming its effectiveness in the improvement of depressive symptoms using less conventional methods. Moreover, actively collected parameters such as depression, sleep, warning signs, and anxiety scores, showed an improved clinical outcome starting from Week 2 of the study.

The strength and direction of the correlations between data collected passively and MADRS score were affected by intra- and interpatient variability during the observation period. Trazodone OAD is a multimodal antidepressant, with recognized activity on depressive symptoms and sleep disturbances associated with MDD. Albeit weak, a relationship between the duration of deep sleep, the duration of light sleep, and MADRS score was observed. A higher sleep score, indicating better sleep quality, was correlated with lower MADRS score, suggesting that better sleep quality may be associated with a better clinical outcome of depression.

During this 8-week study, consistent correlations between active parameters (depression, sleep, warning signs, and anxiety scores, assessed weekly by the patients) and MADRS scores were observed. These correlations suggest that an online questionnaire may be a useful tool for close monitoring of patients, outside of scheduled visits and to allow patients to be engaged, providing direct feedback about therapy. This approach has the additional advantage of being completed by patients at home, without the need for a clinic visit.

The COVID-19 pandemic highlighted the ability of digital technology to replace face-to-face interactions, with schoolchildren, work colleagues, and family members adopting or increasing usage of chats and digital platforms to communicate. The adoption of telemedicine (38), particularly for managing outpatient visits during the pandemic (39) may have significant effects on the increasing use of smartphones, apps, and wearable sensors to monitor health (40). The pandemic is also likely to have affected patients’ willingness to attend busy clinical settings (40, 41), an effect which may continue beyond concerns relating only to COVID-19. With increasing access to smartphones (5, 42), and a willingness by patients to use smartphones to monitor their mental health (6, 7), data collected passively and actively using smartphones and wearable devices are likely to contribute to transforming mental healthcare (43).

The diagnosis and assessment of the severity of symptoms of mental health disorders, such as MDD, are not driven by blood tests, radiologic findings, or electrophysiological measurements (2, 44). Instead, diagnosis and treatment outcome are largely based on clinical evaluations and observations, and patients’ subjective descriptions and recall of data on symptoms, and behavioral and daily life functional changes. (11, 44, 45). This is particularly true for patients with depression, for whom reporting symptoms and feelings experienced over a period of time to the caregiver during a short in-clinic visit may be difficult (44, 45).

The development and increased use of wearable devices (40, 44), as well as adherence and interest in using such devices (43, 45), point to benefits of using digital phenotyping and ePRO alongside traditional clinic visits. Data collected passively will likely have a high degree of accuracy, provided patients are given clear instructions and the device is used correctly. Data collected through regular questionnaires *via* an online platform will not be subject to the recall ability of the patient over a long period of time. Decentralized clinical trials, which enable activities to be carried out at a time and place convenient to the patient, or without any input from the patient beyond wearing and charging a device, reduce patient burden. This is seen as a step toward overcoming a major barrier to recruitment and retention (40, 46). In the same way, it would be hoped that reducing the burden to the patient of monitoring and reporting symptoms would improve accuracy of symptoms’ severity and ease treatment adherence, eventually improving clinical outcomes.

To increase adoption of digital phenotyping and active data collection (*via* apps or web-based platforms), privacy concerns of patients must be assuaged. Knowledge about mobile sensing apps has been found to be key for participants’ comfortability and intention to the use the app (43). Additionally, participants must feel in control of what data are being shared, and with whom. Interestingly, participants who have received mental health treatment have been shown to be more comfortable with sharing data collected *via* remote sensing apps than those who have not received mental health treatment, possibly due to a trusting relationship developed over time with their mental health professionals (43).

Despite the limited number of patients enrolled in this clinical study, we believe that the coherence of the trends observed between the passive and active data collection and the final clinical evaluation, a hybrid approach merits further investigation in larger, controlled clinical trials. Digital tools, when combined with the traditional medical practices and evaluation, could improve patient engagement, and provide early information about the treatment response and tolerability, with the benefit of minimal burden on the patient.

We believe that a major benefit of using passively collected data to track the evolution of depressive symptoms is that the burden on the patient can be minimal, compared to traditional data collection methods. Digital phenotyping, *via* wearable sensors, represents a promising monitoring method to better understand the effect of treatment on the overall functionality of patients and, although limited, the results from this study suggest that further investigation is warranted.

## Data availability statement

The original contributions presented in the study are included in the article/Supplementary material, further inquiries can be directed to the corresponding author.

## Ethics statement

The study received ethical approval from the Etická komise FN Královské Vinohrady (reference number: LEK/04/00/202l) and the Etická komise Research Site s.r.o. (reference number: 219058). Patients provided their written informed consent to participate in this study.

## Author contributions

PL, AB, and ACo contributed to the study conception and design. JČ, SP, and AN contributed to the study management and data collection. AR and ACa reviewed the study results. All authors commented on previous versions of the manuscript. All authors contributed to the article and approved the submitted version.

## Funding

Angelini Pharma S.p.A sponsored this study and supported the writing of the manuscript.

## Conflict of interest

JČ, SP, and AN received principal investigator fees from Angelini Pharma S.p.A. PL, AR, ACo, and ACa are full-time employees of Angelini Pharma S.p.A. AB was a full-time employee of Angelini Pharma S.p.A. at the time of study conduction. The authors declare that this study was sponsored by Angelini Pharma S.p.A. The funder had the following involvement in the study: study design, collection, analysis, interpretation of data, the writing of this article, and the decision to submit it for publication.

## Publisher’s note

All claims expressed in this article are solely those of the authors and do not necessarily represent those of their affiliated organizations, or those of the publisher, the editors and the reviewers. Any product that may be evaluated in this article, or claim that may be made by its manufacturer, is not guaranteed or endorsed by the publisher.
