# Supplementary material for: An Observational Pilot Study using a Digital Phenotyping Approach in Patients with Major Depressive Disorder Treated with Trazodone
Source: Front Psychiatry. 2023 Mar 24;14:1127511. doi: 10.3389/fpsyt.2023.1127511 (PMC10080076; doi:10.3389/fpsyt.2023.1127511)
Supplement: Supplementary file 1 [file Table_1.DOCX]

**Supplementary Table 1: Correlation between passive and active data by study week (mITT population)**

| **Timepoint**  **Passive Parameter**  **Statistics** | **Overall**  **(N = 10)** |
| --- | --- |
| **Study Week 1** |  |
| Distance traveled (m) |  |
| Spearman’s correlation between distance traveled (m) and depression score | -0.0917 |
| Spearman’s correlation between distance traveled (m) and sleep score | -0.3731 |
| Spearman’s correlation between distance traveled (m) and warning signs score | 0.1858 |
| Spearman’s correlation between distance traveled (m) and anxiety score | 0.1166 |
| Spearman’s correlation between distance traveled (m) and medication intake score | 0.5222 |
|  |  |
| Step count |  |
| Spearman’s correlation between step count and depression score | -0.0917 |
| Spearman’s correlation between step count and sleep score | -0.3731 |
| Spearman’s correlation between step count and warning signs score | 0.1858 |
| Spearman’s correlation between step count and anxiety score | 0.1166 |
| Spearman’s correlation between step count and medication intake score | 0.5222 |
|  |  |
| Calories burned (kilocalories) |  |
| Spearman’s Correlation between Calories Burned (kilocalories) and Depression Score | 0.0489 |
| Spearman’s Correlation between Calories Burned (kilocalories) and Sleep Score | -0.2141 |
| Spearman’s Correlation between Calories Burned (kilocalories) and Warning Signs Score | 0.2849 |
| Spearman’s Correlation between Calories Burned (kilocalories) and Anxiety Score | 0.2270 |
| Spearman’s Correlation between Calories Burned (kilocalories) and Medication Intake Score | 0.5222 |
|  |  |
| Duration of deep sleep period (hours) |  |
| Spearman’s correlation between duration of deep sleep period (hours) and depression score | -0.2324 |
| Spearman’s correlation between duration of deep sleep period (hours) and sleep score | -0.6300 |
| Spearman’s correlation between duration of deep sleep period (hours) and warning signs score | -0.0991 |
| Spearman’s correlation between duration of deep sleep period (hours) and anxiety score | -0.0368 |
| Spearman’s correlation between duration of deep sleep period (hours) and medication intake score | -0.0580 |
|  |  |
| Duration of light sleep period (hours) |  |
| Spearman’s correlation between duration of light sleep period (hours) and depression score | 0.5872 |
| Spearman’s correlation between duration of light sleep period (hours) and sleep score | 0.2508 |
| Spearman’s correlation between duration of light sleep period (hours) and warning signs score | 0.1301 |
| Spearman’s correlation between duration of light sleep period (hours) and anxiety score | 0.3620 |
| Spearman’s correlation between duration of light sleep period (hours) and medication intake score | -0.1741 |
|  |  |
| Duration of awake sleep period (hours) |  |
| Spearman’s correlation between duration of awake sleep period (hours) and depression score | 0.3670 |
| Spearman’s correlation between duration of awake sleep period (hours) and sleep score | 0.5872 |
| Spearman’s correlation between duration of awake sleep period (hours) and warning signs score | 0.1053 |
| Spearman’s correlation between duration of awake sleep period (hours) and anxiety score | 0.0675 |
| Spearman’s correlation between duration of awake sleep period (hours) and medication intake score | 0.1741 |
|  |  |
| Wake‑up count |  |
| Spearman’s correlation between wake‑up count and depression score | 0.1433 |
| Spearman’s correlation between wake‑up count and sleep score | 0.6449 |
| Spearman’s correlation between wake‑up count and warning signs score | 0.1830 |
| Spearman’s correlation between wake‑up count and anxiety score | 0.1688 |
| Spearman’s correlation between wake‑up count and medication intake score | 0.0000 |
|  |  |
| Duration of sleep (hours) |  |
| Spearman’s correlation between duration of sleep (hours) and depression score | 0.4710 |
| Spearman’s correlation between duration of sleep (hours) and sleep score | -0.1162 |
| Spearman’s correlation between duration of sleep (hours) and warning signs score | 0.1734 |
| Spearman’s correlation between duration of sleep (hours) and anxiety score | 0.3927 |
| Spearman’s correlation between duration of sleep (hours) and medication intake score | -0.2901 |
|  |  |
| Sleep score |  |
| Spearman’s correlation between sleep score and depression score | 0.1040 |
| Spearman’s correlation between sleep score and sleep score | -0.6055 |
| Spearman’s correlation between sleep score and warning signs score | -0.0434 |
| Spearman’s correlation between sleep score and anxiety score | 0.2086 |
| Spearman’s correlation between sleep score and medication intake score | -0.0580 |
|  |  |
| **Study Week 8** |  |
| Distance traveled (m) |  |
| Spearman’s correlation between distance traveled (m) and depression score | -0.0244 |
| Spearman’s correlation between distance traveled (m) and sleep score | -0.0719 |
| Spearman’s correlation between distance traveled (m) and warning signs score | -0.6145 |
| Spearman’s correlation between distance traveled (m) and anxiety score | 0.1317 |
| Spearman’s correlation between distance traveled (m) and medication intake score | -0.4124 |
|  |  |
| Step count |  |
| Spearman’s correlation between step count and depression score | -0.1952 |
| Spearman’s correlation between step count and sleep score | -0.2036 |
| Spearman’s correlation between step count and warning signs score | -0.6747 |
| Spearman’s correlation between step count and anxiety score | 0.0359 |
| Spearman’s correlation between step count and medication intake score | -0.5774 |
|  |  |
| Calories burned (kilocalories) |  |
| Spearman’s correlation between calories burned (kilocalories) and depression score | 0.5855 |
| Spearman’s correlation between calories burned (kilocalories) and sleep score | 0.7545 |
| Spearman’s correlation between calories burned (kilocalories) and warning signs score | 0.0723 |
| Spearman’s correlation between calories burned (kilocalories) and anxiety score | 0.4311 |
| Spearman’s correlation between calories burned (kilocalories) and medication intake score | 0.4124 |
|  |  |
| Duration of deep sleep period (hours) |  |
| Spearman’s correlation between duration of deep sleep period (hours) and depression score | -0.9271 |
| Spearman’s correlation between duration of deep sleep period (hours) and sleep score | -0.5270 |
| Spearman’s correlation between duration of deep sleep period (hours) and warning signs score | -0.6266 |
| Spearman’s correlation between duration of deep sleep period (hours) and anxiety score | -0.7186 |
| Spearman’s correlation between duration of deep sleep period (hours) and medication intake score | -0.5774 |
|  |  |
| Duration of light sleep period (hours) |  |
| Spearman’s correlation between duration of light sleep period (hours) and depression score | 0.5367 |
| Spearman’s correlation between duration of light sleep period (hours) and sleep score | 0.7904 |
| Spearman’s correlation between duration of light sleep period (hours) and warning signs score | 0.5784 |
| Spearman’s correlation between duration of light sleep period (hours) and anxiety score | 0.5629 |
| Spearman’s correlation between duration of light sleep period (hours) and medication intake score | 0.5774 |
|  |  |
| Duration of awake sleep period (hours) |  |
| Spearman’s correlation between duration of awake sleep period (hours) and depression score | -0.1220 |
| Spearman’s correlation between duration of awake sleep period (hours) and sleep score | 0.3713 |
| Spearman’s correlation between duration of awake sleep period (hours) and warning signs score | 0.2289 |
| Spearman’s correlation between duration of awake sleep period (hours) and anxiety score | -0.0240 |
| Spearman’s correlation between duration of awake sleep period (hours) and medication intake score | 0.5774 |
|  |  |
| Wake‑up count |  |
| Spearman’s correlation between wake‑up count and depression score | 0.1482 |
| Spearman’s correlation between wake‑up count and sleep score | 0.1697 |
| Spearman’s correlation between wake‑up count and warning signs score | 0.0305 |
| Spearman’s correlation between wake‑up count and anxiety score | -0.1394 |
| Spearman’s correlation between wake‑up count and medication intake score | 0.5843 |
|  |  |
| Duration of sleep (hours) |  |
| Spearman’s correlation between duration of sleep (hours) and depression score | -0.1220 |
| Spearman’s correlation between duration of sleep (hours) and sleep score | 0.4192 |
| Spearman’s correlation between duration of sleep (hours) and warning signs score | 0.1928 |
| Spearman’s correlation between duration of sleep (hours) and anxiety score | 0.5629 |
| Spearman’s correlation between duration of sleep (hours) and medication intake score | -0.2474 |
|  |  |
| Sleep score |  |
| Spearman’s correlation between sleep score and depression score | -0.6099 |
| Spearman’s correlation between sleep score and sleep score | -0.0719 |
| Spearman’s correlation between sleep score and warning signs score | -0.3856 |
| Spearman’s correlation between sleep score and anxiety score | -0.3114 |
| Spearman’s correlation between sleep score and medication intake score | -0.5774 |
